# The Utilization of PRAME in the Diagnosis, Prognosis, and Treatment of Melanoma

**DOI:** 10.3390/cells13201740

**Published:** 2024-10-20

**Authors:** Samuel L. Blount, Xiaochen Liu, Jeffrey D. McBride

**Affiliations:** 1 College of Medicine, University of Oklahoma Health Sciences Center, Oklahoma City, OK 73104, USA; samuel-blount-1@ouhsc.edu; 2 Department of Dermatology, University of Oklahoma Health Sciences Center, Oklahoma City, OK 73104, USA; xiaochen-liu@ouhsc.edu

**Keywords:** melanoma, PRAME, cell biology, cancer, immunotherapy

## Abstract

Melanoma, a deadly form of skin cancer, has seen improved survival rates due to advances in diagnosis and treatment, yet the need for further improvement remains critical. Tumor-associated antigens, such as PRAME (Preferentially Expressed Antigen in Melanoma), offer promising avenues for enhanced diagnostic precision, prognostic assessment, and targeted immunotherapy. PRAME, a cancer testis antigen, is selectively expressed in various cancers, including melanoma, and plays a key role in promoting tumorigenesis through inhibition of retinoic acid signaling, epithelial-to-mesenchymal transition, and immune evasion. This review explores the diagnostic utility of PRAME in distinguishing melanoma from benign nevi, its prognostic value in aggressive melanoma subtypes, and its potential as a therapeutic target in cancer vaccines and adoptive T-cell therapies. While PRAME-targeted therapies face challenges such as tumor heterogeneity and immune suppression, ongoing research aims to overcome these barriers, offering hope for more effective melanoma treatments.

## 1. Introduction

Melanoma, known to develop in part from UV radiation damage of melanocyte DNA or other inherited or acquired mutations, is one of the deadliest forms of skin cancer. The American Cancer Society estimates that in the US, approximately 100,640 new cases of melanoma will be diagnosed in 2024. Of these, 8290 are expected to die from their cancer. Additionally, melanoma incidence rates are consistently rising. The statistics, however, are not all discouraging. Death rates secondary to melanoma have declined by 6% to 7% per year from 2013 to 2017. The reductions in death rates can be explained by the improvements in diagnosis and treatment. Over the past three decades, several paradigm-shifting therapeutics have been developed for the treatment of cancer, including immune checkpoint inhibitors (ICIs), BRAF and MEK targeted therapies, and cancer vaccines [1]. ICIs, for example, were demonstrated to extend median overall survival from 11.2 months in 2013 to 72.1 months in the CheckMate 067 trial in 2021 [2,3]. Improvements in melanoma screening and rapidity of diagnosis have also contributed to the reduction in death rates. Despite these improvements, the number of deaths remains unacceptably high. Further advancements in melanoma diagnosis and treatment are critical.

A particular area of research that poses promising solutions for the diagnosis and treatment of melanoma is the identification of antigen biomarkers. Specifically, investigators are searching for tumor-associated antigens (TAAs), proteins expressed by tumor cells, exclusively or at least in higher quantities, as compared to somatic cells. The identification of antigen biomarkers could be utilized in the diagnostic process through the development of immunohistochemistry. These antibodies recognize specific proteins and would provide high utility for pathologists with challenging melanoma cases. Moreover, TAAs would make excellent targets for immune-based therapies. Utilizing T-cells or cancer vaccines to target specific TAAs could provide maximal destruction of tumor cells while minimizing damage to normal tissues. A current TAA being studied in these capacities is PRAME, or Preferentially Expressed Antigen in Melanoma. This paper seeks to explore the utilization of PRAME in different clinical contexts for the management of melanoma, namely diagnostic, prognostic, and therapeutic contexts.

## 2. The Biology of PRAME and Other Cancer Testis Antigens

### 2.1. Overview of the PRAME Protein

PRreferentially expressed Antigen in MElanoma (PRAME) is a 509-amino-acid protein found in the nuclear and cytoplasmic space of several different cell types in the human body. The gene encoding PRAME is found on chromosome 22 (22q11.22), embedded among several genes encoding immunoglobulin proteins [4]. PRAME is a member of the cancer testis antigen (CTA) family. CTAs are proteins typically found in germline cells of the ovaries and testis and are thought to play a role in gametogenesis [4]. These antigens have also been found in various hematologic and solid tumor malignancies. Many CTAs are present on human leukocyte antigen class 1 (HLA-1) molecules in cancer cells, allowing for recognition by T-lymphocytes [4,5,6]. PRAME was first identified by H. Ideka et al. in melanoma cell lines in 1997 while investigating potential therapeutic targets for immunotherapy [7].

### 2.2. Biology of Cancer Testis Antigens

Cutting-edge technologies like cancer vaccines and T-cell therapy are exciting due to their utilization of the immune system to find and destroy tumor cells with high selectivity. To continue developing better immunotherapies, investigators must identify immunogenic antigens that are selectively expressed on the HLA-1 molecules of cancer cells and not normal cells. These specific antigens in tumor cells are often called tumor-associated antigens (TAAs). In hopes of discovering TAAs, researchers led by Lloyd J Old performed autologous typing experiments with various tumor cells and found a new class of TAAs he called cancer testis antigens (CTAs) [8,9]. Twenty-five years later, autologous typing techniques were superseded by serological analysis of cDNA expression libraries (SEREX), which led to a rapid expansion of the known CTAs [6,8]. These antigens are called cancer testis antigens because of their expression in both tumor cells and testis germline cells. Today, the CTA family includes over 200 distinct proteins [8,10]. CTAs can be divided into two subclasses by their chromosomal location. One class represents genes found on X-chromosomes, and the other represents genes found on either autosomes or Y-chromosomes [8,10]. In adult somatic tissues, CTA expression is typically restricted to germline cells of the testis, ovaries, and placenta [6,8,10]. However, recent experiments suggest that CTAs such as PRAME are also found in the adrenal and endometrial tissues [4,11]. CTAs are known to be involved in the differentiation and proliferation of germline cells, but the biological mechanisms remain to be fully elucidated [8,11].

### 2.3. The Role of PRAME and Other Cancer Testis Antigens in Tumorigenesis

**Figure 1:**
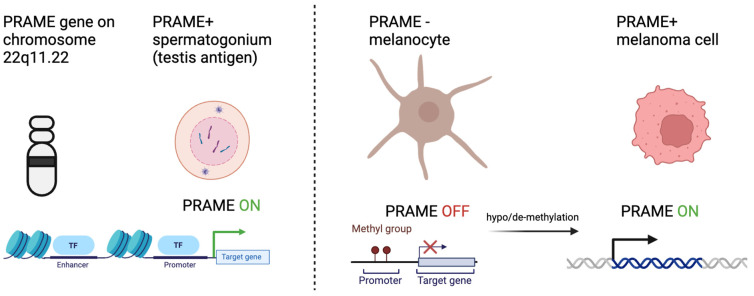
PRAME expression in early progenitor cells and reactivation in melanoma cells. PRAME is located on chromosome 22 and normally expressed in developing gamete cells. PRAME expression is associated with an undifferentiated, proliferative state in cells. Methylation at the promoter region inhibits its expression in benign melanocytes. Via mechanisms not fully yet known, PRAME expression is reactivated in almost all melanoma types, in part due to demethylation at the PRAME promoter.

Many plausible theories have been developed that attempt to explain the process of somatic cells transitioning to tumor cells. One such theory is that certain silenced genes involved in gametogenesis become activated, resulting in a highly proliferative, apoptosis-resistant, and migratory phenotype implicated in cancer [8]. Given the role of PRAME in gametogenesis, its increased expression is thought to promote tumorigenesis through inhibition of retinoic acid signaling, promotion of epithelial to mesenchymal transition, and promotion of a pro-tumor microenvironment. In testis cells, for example, when a gametocyte differentiates into a mature spermatocyte, epigenetic programs methylate promoter regions of DNA that encode proteins involved in gametogenesis. Once the promotor region is methylated, transcription factors can no longer bind, resulting in decreased genetic expression [12]. In all somatic tissues, excluding the placenta and testis germline cells, the promoter regions of genes encoding CTAs are hypermethylated. In the germline cells, by contrast, the promoter regions are hypomethylated. Interestingly, several studies exploring CTA and PRAME biology in cancer note similar hypomethylation patterns for CTA genes. Somatic tissue with methylated promoter regions can become hypomethylated, which increases the expression of CTA product proteins that promote tumor phenotypes [4,13]. These methylation patterns suggest that epigenetic modifications of CTA genes occur in somatic cells, promoting the transition to cancerous phenotypes (Figure 1).

**Figure 2:**
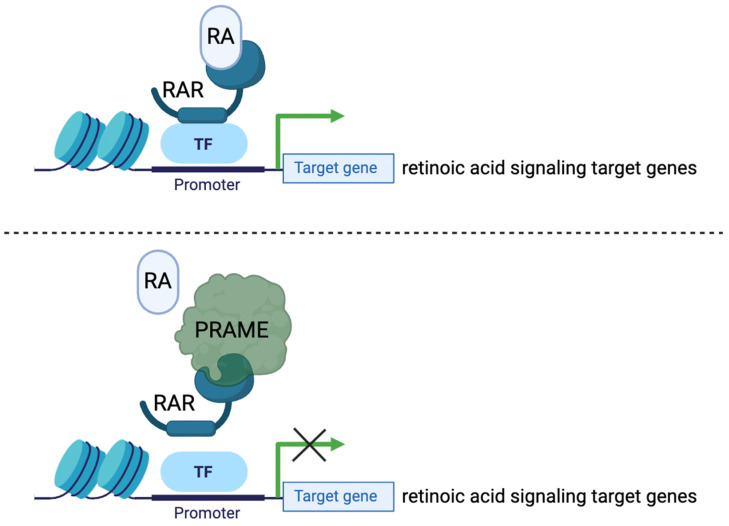
PRAME inhibits binding of retinoic acid (RA)–retinoic acid receptor (RAR), inhibiting target gene expression. RA binds to nuclear hormone receptor RAR to induce expression of retinoic acid signaling target genes. PRAME binds to RAR to inhibit the expression of the target genes. Switching OFF of these target genes results in a more proliferative, migratory, and less-differentiated cell state (characteristics that are displayed in melanoma cells).

The protein PRAME has been identified to promote a cancerous phenotype through its involvement with retinoic acid signaling pathways (Figure 2). Retinoic acid signaling (RAS) is a biochemical pathway involved in the regulation of cell differentiation, proliferation arrest, and apoptosis [5,14]. In normal cells, retinoic acid (RA), commonly known as vitamin A, binds to retinoic acid receptor proteins α, β, or γ, which then activate genetic programs involved in cell cycle regulation and growth arrest [15]. Normal RAS has anti-tumor effects. Remarkably, activation of RAS through a form of exogenous RA called all-trans retinoic acid (ATRA) has been demonstrated to inhibit the growth and progression of acute promyelocytic leukemia (APL) [16]. Though not entirely established, it is thought that PRAME acts as a dominant repressor of RAS through competitive binding to RA receptors. PRAME prevents RA from binding properly, resulting in the downregulation of target genes involved in growth arrest [4,5]. This is a plausible explanation for the high expression of PRAME in tumor cells with highly proliferative phenotypes.

PRAME, among other CTAs, has been shown to increase the metastatic potential of cancer cells. Several studies have found that tumor cells of metastatic lesions exhibit higher expression levels of CTAs than the cells of the primary tumor site, suggesting they may play a role in cancer metastasis. Researchers have determined one plausible mechanism for this increased metastatic potential. The CTAs PRAME, CT45A1, and MAGEC2 are known to induce epithelial-to-mesenchymal transitions (EMTs) in tumor cells. EMT is a process by which somatic cells lose their cell-to-cell adhesion properties and surface polarity, leading to increased cell motility and migratory potential [8,17,18,19]. How PRAME induces EMT is not understood, but the mechanism could be similar to the protein CT45A1, which induces EMT in osteosarcoma cells through the β-catenin pathway [18,19]. The third understood role of PRAME in tumorigenesis is in its promotion of a pro-tumor microenvironment by increasing tumor “coldness”. Cold tumors are cancer cells that evade the immune system through downregulation of antigen-presenting molecules and increase production of immune checkpoint molecules. These “tumor-cold” environments limit the efficacy of immunotherapeutics. PRAME was shown to be involved in this process in a breast cancer study. Investigators A. Naik et al. found that silencing of PRAME genes in MDA-MB-468 breast cancer cells reduced the expression of immune checkpoint molecules and promoted cancer cytolysis by T-cells. In the same study, overexpression of PRAME was found to decrease the activation of T-cells and reduce the cytolytic potential [20]. Further research must be carried out to better understand the role of PRAME in cancer through its involvement in RAS, EMT promotion, and establishment of a tumor-cold microenvironment.

## 3. Diagnostic Potential of PRAME in Melanoma

### 3.1. Current Histopathologic Techniques Used in Melanoma Diagnosis

Melanoma is a cancer of the melanocytes. Many melanomas bear similar features, clinically and pathologically, to benign pigmented or dysplastic lesions, leading to an often-challenging differential diagnosis. Clinically, dermatologists utilize the ABCDE criteria for distinguishing melanomas from benign nevi [21]. These criteria include **A**symmetry, **B**order irregularity, **C**olor change, **D**iameter of greater than 6 mm, and **E**volution [21,22]. If making a confident diagnosis is difficult, clinicians can biopsy the lesions for pathology to review. Followed by staining with hematoxylin and eosin (H&E), pathologists look for irregular morphologic features like asymmetry, poor circumscription, cytologic atypia, mitotic activity, and failure of maturation with descent [21]. Despite careful analysis of these features, many difficult-to-diagnose cases persist. Diagnosing melanomas with high specificity and sensitivity is essential because incorrect or delayed diagnoses can result in unnecessary treatment or delays in necessary treatment. Immunostaining for a variety of protein markers is often used to increase diagnostic accuracy. Common IHC stains used in the diagnosis and staging of melanomas are S-100, SOX10, Melan-A, and HMB-45 [23,24]. S100 is not specific to melanocytes. Sox-10 and Melan-A confirm melanocytic origins, but do not capture a malignant phenotype. Pathologists may also utilize cytogenic tests like fluorescence in situ hybridization (FISH) or single-nucleotide polymorphism array (SNP) to examine the DNA from biopsied tissue samples to identify genetic aberrations [21]. This allows for the identification of genetic aberrations implicated in cancer. Cytogenic testing typically yields more accurate diagnoses; however, there is no perfect test, and final diagnosis relies on clinical, pathologic, and molecular correlation. However, the cost and availability of molecular testing often result in less frequent or restricted use of these tests. Because of this, pathologists favor the widely available immunostaining methods for melanoma diagnosis. Continued research is being conducted to identify candidate antigens for IHC. PRAME has become a strong antigen of interest.

### 3.2. Utilization of PRAME in Melanoma Diagnosis

Melanocytes are found in a myriad of locations throughout the body, from the lymph nodes to the epidermis. Thus, melanomas are diverse in their location, presentation, and prognosis. The list of melanoma subtypes includes superficial spreading melanoma, lentigo maligna, acral melanoma, desmoplastic melanoma, non-desmoplastic melanoma, nevoid melanoma, uveal melanoma, spitzoid melanoma, and mucosal melanoma [25,26]. Each of the different melanoma subtypes has a benign lesion counterpart with similar clinical characteristics. Many studies have been performed to explore the frequency of PRAME expression in melanoma compared with benign tumors. The research has shown PRAME to be a highly selective and sensitive marker in melanoma diagnosis. Research by Lezcano et al. found that of 100 lesions confirmed to be metastatic melanoma, 92 were diffusely PRAME-positive (92%). Similarly, a sample of 155 primary cutaneous melanomas, excluding desmoplastic melanomas, showed PRAME positivity in 92% of the cases [27]. These data highlight the sensitivity of PRAME staining. In the same study, specificity was measured by PRAME staining of 140 benign cutaneous and nodal melanocytic nevi. Of these nevi, 86.4% were PRAME-negative [27]. A similar study performed by Andrea Ronchi et al. utilized immunocytochemistry (ICC) methods to explore PRAME positivity in melanomas. PRAME positivity was observed in 85.4% of 48 cutaneous melanoma metastases. The sensitivities of PRAME were not as significant as S100 (100%), Melan-A (97.9%), SOX10 (100%) and HMB45 (89.6%) [24]. Though PRAME IHC is less sensitive than the other IHC stains marking melanocytes, it may offer greater diagnostic utility by being much more specific for melanoma. PRAME is selectively expressed in various malignancies and limitedly expressed in benign cells. When staining with Melan-A and SOX10, benign and malignant melanocytes are stained, making margin assessment difficult. Staining with PRAME allows for clearer margin assessment and better visualization of malignant melanocytes. For these reasons, PRAME IHC offers unique clinical utility by providing more accurate margin assessment and Breslow thickness, both of which are important in staging and clinical management of melanoma [27]. Additional studies have evaluated the sensitivity of PRAME IHC compared with other ancillary cytogenic testing like FISH and SNP-Array. A separate study conducted by Cecilia Lezcano revealed that out of a cohort of 110 diagnostically challenging melanocytic tumors, there was a concordance of 90% between cytogenic testing and PRAME IHC as well as a concordance of 92.7% between PRAME IHC and the final diagnosis. High concordance with cytogenic testing is a testament to the diagnostic utility of PRAME.

BRAF V600E and V600K mutations are commonly associated with driving melanomagenesis [28]; however, we currently do not have a good understanding of PRAME expression in BRAF V600E versus V600K melanoma. The higher mutational burden and distinct immune responses observed in V600K melanomas suggest that PRAME expression could potentially differ from other subtypes. It is well established that desmoplastic melanomas typically exhibit lower PRAME expression [27]. The increased mutational load in V600K melanomas may indicate a more immunogenic environment, which could influence PRAME expression levels, but this relationship needs to be studied further.

As previously mentioned, melanocytes can be present in the lymph nodes. The benign collections of melanocytes in the lymph nodes are called nodal nevi. Nodal nevi are morphologically similar to melanoma lymph node metastases, making the differential diagnosis challenging. Lezcano et al. performed a study evaluating the expression of PRAME in nodal nevi compared to melanoma metastases. They found that of 30 nodal nevi, 0% were PRAME-positive. These data demonstrate remarkable specificity. Furthermore, of the 15 melanoma metastases in the lymph nodes, 100% were PRAME-positive [29]. Further research should be carried out to confirm this high sensitivity of PRAME IHC to improve the diagnostic accuracy of lymph node biopsies in melanoma patients. Another challenging class of cases in clinical practice is acral lesions, which are pigmented lesions on the soles, palms, knees, elbows, and nails. Benign acral lesions have similar pathologic and morphologic characteristics under microscopy compared with melanomas, necessitating IHC and ancillary cytogenic testing to confirm diagnoses. PRAME IHC was found to be positive in 87.1% of malignant acral lesions and faintly expressed or not at all expressed in 82.5% of benign lesions in a study conducted by Giacomo Santandrea et al. This research suggests a sensitivity of 87.1% and a specificity of 82.5% for PRAME IHC [30]. One of the authors of this manuscript (McBride) found that, in a cohort at the Cleveland Clinic, 100% of acral melanomas (*n* = 10) were positive for PRAME IHC, and, of the benign, dysplastic, and spitz acral nevi (*n* = 20), all were negative for PRAME [31]. Studies have revealed that PRAME is expressed in the majority of melanomas, including both in situ and invasive types, but is rarely detected in benign nevi and melanocytic lesions [27,32]. These findings support PRAME as a biomarker for distinguishing malignant from benign melanocytic lesions. Specifically, PRAME is more commonly expressed in invasive melanomas compared to benign nevi and in situ melanomas, making it a valuable tool in differentiating these stages of melanoma progression [25,33,34]. Having an accurate IHC stain like PRAME to aid in the diagnosis of acral lesions is extremely useful and should be utilized more often in clinical practice. However, inconsistencies in expression among certain melanoma subtypes can be significant barriers to universal interpretation; PRAME staining must always be taken within the entire context of the neoplasm, along with clinical and, if available, molecular data. Two diagnostically challenging melanocytic proliferations are desmoplastic and spitzoid melanomas. Desmoplastic melanoma, an invasive, neurotropic malignancy, was found to have PRAME positivity in only 35% of cases [27]. However, the gold standard is only the labeled diagnosis of “desmoplastic melanoma”, and PRAME expression in the PRAME+ desmoplastic melanomas may be signaling something fundamentally different about these desmoplastic melanoma types. Spitzoid lesions can be difficult to diagnose on H&E alone and often need additional immunohistochemical and molecular tests as well, especially due to the clinical and pathological similarities between spitz nevi, atypical spitz tumors and spitzoid melanomas [4]. Several studies have identified PRAME IHC to be a useful tool in aiding in the diagnosis of spitzoid melanomas [35]. Stephen Koh’s lab found PRAME positivity in 82% of spitzoid melanomas and only 20% of benign spitz nevi. Conversely, A. Alomari identified PRAME positivity as a possible diagnostic pitfall in spitzoid melanoma diagnosis due to the cases of diffusely positive benign spitzoid lesions, which are likely just in a proliferative state [36]. PRAME correlates highly with the results of molecular testing in melanoma, with high agreement [37]. The strengths and limitations in the sensitivity and specificity of PRAME IHC should continue to be researched. If properly understood, PRAME IHC could yield greater diagnostic utility for future challenging diagnoses. Additionally, the differences in gene expression and prognosis in PRAME+ versus PRAME- melanomas should be studied further. The utility of PRAME extends beyond diagnosis, holding prognostic implications as well as therapeutic potential.

PRAME has utility in detecting melanoma microsatellites as well [38]. Because PRAME is positive in almost all melanoma cells, in PRAME+ melanomas, even small microsatellites can be detected, and would otherwise be at risk of being missed on scanning H&E. Thus, PRAME serves as a powerful tool to enhance the sensitivity of detection in melanoma diagnosis.

In a proof-of-concept study, the expression of PRAME was quantified digitally along with the expression of Sox-10, and a PRAME index was developed. This PRAME index showed a sensitivity of 70% and a specificity of 100% in differentiating melanomas from benign melanocyte lesions [39]. This was on par with manual qualitative methods. As dermatopathology digitalization becomes more widespread across pathology practices, this will likely prove to be a useful diagnostic, and efficient, tool for quantifying PRAME expression in melanomas.

### 3.3. PRAME as a Prognostic Biomarker in Melanoma

PRAME expression has been shown in numerous studies to be highly associated with an unfavorable melanoma prognosis. PRAME, as its name implies, was first identified in melanoma cells and has been demonstrated to increase metastatic potential through repression of RAS, promotion of EMT, and promotion of an immunologically “cold” tumor microenvironment (TME) [4,8,17]. These traits all help to explain the poorer prognosis observed in melanomas and other malignancies with elevated PRAME expression [40,41,42,43,44,45] (Table 1). Most of the prognostic research papers are retrospective studies in uveal melanomas (UMs). UMs are malignancies of the melanocytes within the ocular tissue/uveal tissue and can be stratified into two subclasses by gene expression profiling (GEP). Class 1 UMs have low metastatic risk, and Class 2 UMs have high metastatic risk [46,47]. A retrospective study performed by Amy Schefler et al. (*n* = 148 patients) involving multiple ocular oncology centers found positive PRAME expression to be associated with increased basal diameter, worse GEP values, and increased metastatic risk [47]. A similar analysis by Matthew Field demonstrated higher PRAME positivity in Class 2 UMs compared to Class 1 [40]. Given that Class 2 UMs have greater metastatic potential, these data hold prognostic implications. In the same study, PRAME-positive UMs were found to metastasize more rapidly than the lesions with minimal PRAME expression [46]. This corroborates the role of PRAME in promoting the EMT phenotype, whereby cells lose their surface polarity and gain motility [17]. The prognostic effects of PRAME have also been explored in mucosal melanomas. Out of a sample of 29 mucosal melanomas, 20 (83.3%) had high PRAME expression, correlating to a worse prognosis overall [41]. Though these studies suggest a strong correlation between PRAME positivity and poorer prognosis, an analysis by Parra et al. found no significant difference between PRAME expression in primary cutaneous versus metastatic melanoma; this is not surprising, as PRAME levels would be expected to remain high whether or not we are looking at primary melanoma or metastatic cells [48]. Further research must be conducted to explore the possible prognostic implications of PRAME in early melanomas versus late and in combination with other antigens in all melanoma subtypes.

### 3.4. PRAME as a Prognostic Biomarker in Other Cancer Types

PRAME has proven to be a versatile biomarker, correlating to poorer prognosis in tumor cells expressing it. One such cancer class is breast cancer, where several studies have found increased PRAME expression to be associated with worse outcomes. In 2022 alone, 287,850 US women were diagnosed with invasive breast cancer. Of these women, 43,250 died from their disease [49]. A study analyzing Kaplan–Meier survival curves found that of 295 primary breast cancer lesions, PRAME expression was associated with decreased overall survival and increased rates of metastasis [42]. The exact mechanism explaining how PRAME promotes these outcomes in breast cancer patients is not fully understood, but some studies suggest it stems from PRAME’s involvement in the promotion of an EMT phenotype [17]. PRAME expression is also associated with decreased survival and poorer outcomes in sarcoma patients. A sarcoma is a malignancy of the bone and soft tissues, encompassing a broad range of pathologies. Multiple sarcoma subtypes have exhibited CTA expression, including myxoid liposarcomas, synovial sarcomas, chondrosarcomas, and osteosarcomas [50,51]. A study performed by Roszik et al. found PRAME expression to be associated with lower expression of antigen-presenting protein beta2microglubulin (B2M), providing a biochemical explanation for how PRAME promotes an immunologically cold TME [50]. Cancers that downregulate antigen-presenting molecules are less recognizable by the immune system, resulting in unhindered growth. Research by Iura et al. found similar results. Of 93 confirmed cases of myxoid liposarcomas, a sarcoma subtype with classically poorer prognoses, 90% were PRAME-positive. In the less aggressive dedifferentiated and well-differentiated liposarcomas, only 43% and 9% expressed PRAME, respectively [43]. Studies like these display the promising uses of PRAME in the prognostication of cancer. It should be noted that the role of PRAME in the prognosis of hematologic malignancies, by contrast, is still inconclusive. There are three main categories of hematologic malignancies—leukemias of the leukocytes, lymphomas of the lymphocytes, and myelomas of the plasma cells [45]. Multiple studies have found PRAME to be associated with a poor prognosis in patients with diffuse large B-cell lymphoma, Hodgkin’s lymphoma, multiple myeloma, and chronic leukemia through the promotion of drug resistance and disease progression [45]. Given that increased PRAME expression is associated with poorer prognosis in other cancers, these data are not surprising. The convoluted nature of PRAME in hematologic malignancies comes from research citing the opposite effects. For example, Tajeddine et al. induced overexpression of PRAME in leukemia cell lines and found decreased tumorigenicity by way of induction of caspase-independent cell death in vitro and reduction in the levels of proteins inhibiting apoptosis (Hsp27, p21, and S100A4) in vivo [52]. Another study that contradicts the association of increased PRAME expression with poorer prognosis is the work by Zhang et al., which found overexpression of PRAME to predict better overall outcomes in pediatric B-cell acute lymphoblastic leukemia (ALL) [44]. The contradiction in the literature emphasizes the importance of continued research on the use of PRAME in the prognostication of hematologic malignancies.

As previously discussed, many studies cite the positive association between PRAME expression and the prognosis of individual cancer types. Study designs like these are highly useful and necessary, but the utilization of meta-analysis techniques to evaluate the prognostic implications of PRAME on a broader scale, irrespective of cancer type, provides a unique understanding of the potential of PRAME. One such analysis by Jiaqiang Li et al. assesses the association of PRAME expression with cancer prognosis in 2421 patients. In the study, the parameters of disease-free survival (DFS), progression-free survival (PFS), metastasis-free survival (MFS), and overall survival (OS) were evaluated using both fixed-effect and random-effect models. PRAME expression was shown to be positively associated with all these values. The data showed reductions in DFS (*p* < 0.001), PFS (*p* = 0.042), MFS (*p* = 0.034), and OS (*p* < 0.001) which were all statistically significant (*p* ≤ 0.05). Additionally, increased PRAME expression was found to be associated with advanced clinical stages (III–IV) as well as positive lymph node metastases [53]. More meta-analyses looking at prognostic effects irrespective of cancer type should be conducted for stronger evidence in utilizing PRAME staining in clinical practice.

## 4. PRAME as a Therapeutic Target in Immunotherapy

### 4.1. Overview of Immunotherapy

Despite advances in surgical techniques, chemotherapeutics, and radiation therapies, cancer continues to kill numerous patients each year. Overall survival for cancer patients has improved, but these patients are still subject to metastasis and recurrence. The difficulties in treating cancer lie in the similarities between somatic cells and cancer cells. Cancer cells are almost structurally and genetically identical to their somatic counterparts but with upregulated genetic programming resulting in malignant phenotypes such as immune evasion, replicative immortality, tumor-promoting inflammation, sustained proliferation, and evasion of cell death [54]. Chemo and radiation therapies are excellent at destroying tumor cells, but they also damage healthy tissues in the process. The real difficulty is treating the malignant cells while avoiding harming the somatic cells. This need for well-tolerated treatments with robust clinical responses inspired research into a new, remarkably selective cancer therapeutic: immunotherapy. The first immunotherapies to become FDA-approved were the anti-tumor cytokines interferon-alpha 2 (IFN-a2) and interleukin-2 (IL-2), molecules that stimulate T-cell proliferation [55]. In 2014, more than 20 years later, the FDA approved a new class of cancer therapeutics called immune checkpoint inhibitors (ICIs). ICIs are antibodies against the immune checkpoint molecules Cytotoxic T-Lymphocyte-Associated Antigen 4 (CTLA-4) and Programed Death Ligand 1 (PD-L1), preventing cancer cells from deactivating T-cells [5,8,55]. The more recent therapies that have been developed are the use of genetically engineered T-cells (CAR-T) and ex vivo expansion of circulating antigen-specific T-cells, which work by programming T-lymphocytes to express a surface antigen receptor recognizing a tumor-specific antigen. Crucial to developing T-cell-based immunotherapies is the identification of tumor-selective antigens, those presented by HLA-class 1 molecules of tumor cells and not somatic cells. In recent years, multiple CTAs, including PRAME, have been evaluated as potential targets for immunotherapy as they are selectively presented on the HLA-1 surface proteins of tumor cells. Currently, PRAME is being researched as a target for immunotherapy for cancer vaccines and T-cell-based therapies.

### 4.2. PRAME as a Target in Cancer Vaccines

Cancer vaccines work by administering a synthesized antigen or nucleic acid sequence with adjuvant molecules to mount an immune response against tumors expressing the antigen. The mechanisms are similar to viral vaccines but cancer vaccines are used therapeutically instead of prophylactically [56]. The cancer vaccine technologies currently being utilized are peptide vaccines, dendritic cell vaccines, and nucleic acid vaccines. CTA proteins are prime targets for peptide-based cancer vaccines due to their tumor selectivity. Trials exploring the efficacy of MAGE-A and NY-ESO-1 vaccines are ongoing, many eliciting both cellular and humoral responses [8]. For instance, data from phase I and II trials for these CTAs have shown antibody, CD4+, and CD8+ responses with minimal off-target toxicities [5,10]. However, when the trials progressed to phase III, different conclusions emerged. Two phase III clinical trials evaluating the efficacy of MAGE-A3 peptide-based vaccines in the treatment of non-small-cell lung cancer (NSCLC) (*n* = 2272 patients) and melanoma (*n* = 1345 patients) have been performed. Both studies were terminated from a lack of improvement in disease-free survival in treatment groups compared with controls [10]. A recurring situation in the field of CTA cancer vaccines has been the problem of little efficacy being demonstrated in clinical trials. Similarly, PRAME vaccine trials have not progressed past phase I trials. In murine samples treated with PRAME antigen vaccines, CD4+ and CD8+ T-cell responses were observed which encouraged the escalation to primate model studies. Phase I studies in primates, metastatic melanoma patients, and NSCLC patients found antibody responses but limited CD4+ and no CD8+ responses [57,58]. The lack of CD8+ responses has discouraged the escalation to phase II or III clinical trials. CD8+ responses, as previously discussed, are crucial for the destruction of tumor cells. Several plausible explanations for the observed lack of efficacy exist and will be discussed in the limitations section. In brief, a melanoma patient’s immune cells may have become tolerant to PRAME as an antigen to stimulate killing of tumor cells. A more important strategy would be to hone or re-induce killer cells to PRAME+ melanoma cells. To improve the CD8+ response, investigators are exploring the use of polyvalent vaccines that target multiple antigens simultaneously. Research by Weber et al. found that a polyvalent vaccine targeting PRAME and prostate-specific membrane antigen induced expansion of CD8+ T-cells recognizing PRAME and PSMA in 15 out of 24 prostate cancer patients. In addition to these findings, the researchers found that seven patients showed stable disease for six months or longer [59]. Further research must be performed to expand upon this knowledge of polyvalent vaccine technologies targeting CTAs.

### 4.3. PRAME as a Target in Adoptive T-Cell Therapy

T-cell therapy can be described as the utilization of cytotoxic T-lymphocytes (CTLs or CD8+ cells) to target and destroy tumor cells expressing specific antigens. CTLs circulate through the body and screen for aberrant gene products expressed on HLA-1 molecules. Circulating CTLs with unique T-cell receptors (TCRs) recognize specific antigen epitopes. When the CTL identifies a cell with a concerning antigen, it releases granzymes and perforin to destroy the cell harboring the antigen [60,61]. T-cells can recognize foreign antigens with remarkable specificity, posing an exciting solution to the grandest challenge to cancer therapeutics, which is maximizing tumor destruction while minimizing harm to somatic tissues. Chemotherapeutics are efficient at harming cancer cells, but they also have significant side effects that cannot be ignored. The remarkable ability of the cell-mediated immune response to recognize foreign invaders while limiting harm to itself has led researchers to develop therapies utilizing CTLs in cancer treatment.

The two classes of adoptive cell transfer, commonly known as T-cell therapy, being researched are ex vivo expansion of tumor-infiltrating lymphocytes (TILs) and chimeric antigen receptor (CAR) T-cell therapy (Table 2) [5,60]. Ex vivo expansion of TILs is a process where lymphocytes that have successfully infiltrated and recognized TAAs on tumor cells are removed from patients, expanded through treatment with T-cell growth factors IL-2, IL-7 or IL-21, and, once reaching sufficient population size, readministered to the patient. This process was first developed by Rosenberg et al. in 1988 for the treatment of metastatic melanoma patients. They found potent clinical response and durable regression in 11 out of 20 patients [62]. In the study, TILs from melanoma tumors were harvested following surgical resection. Once collected, the TILs were selected to recognize specific tumor antigens. The selected TILs were then expanded and infused into patients with adjuvant IL-2 [62]. Since Rosenberg pioneered ACT with TILs, it has been researched in both solid organ tumors and hematologic cancers. A recent meta-analysis by Dafni et al. investigating TIL therapy for melanoma patients found an objective response rate of 41% in 410 melanoma patients previously treated with chemotherapy or radiation [61]. Because of the aggressive nature of cancer, response rates in any form are encouraging. Thus, the 41% response rate for TIL therapy is a promising result. To improve the strength and durability of responses, several studies have experimented with TILs recognizing tumors expressing CTAs. With proteasome cleavage analysis of PRAME, four high-affinity HLA-A*201-restricted epitopes were identified to be recognizable by T-cells [63]. Two different labs have searched for circulating TILs recognizing these four PRAME-restricted epitopes in melanoma and AML patients. The researchers found these PRAME-specific TILs in 36% of melanoma patients and 70% of AML patients [64,65]. Trials exploring the efficacy of ACT with TILs targeting PRAME epitopes are ongoing with encouraging preclinical results, but substantial clinical data are lacking.

The other type of ACT is called CAR T-cell therapy. CAR T-cell therapy involves genetically modifying the receptors of T-cells to recognize specific antigen epitopes with high affinity. CAR T-cells function similarly to endogenous CTLs, releasing granzymes and perforin in cells identified as foreign. What makes CAR T-cell therapy unique is that its chimeric antigen receptor recognizes antigens irrespective of expression on HLA-1 molecules [66]. Chimeric antigen receptors can be programmed to recognize any antigen on the surface of cells, resulting in a powerful anti-tumor response [66]. Since its development in 1989, six CAR T-cell therapies have been approved by the FDA. Four of the therapies target cluster of differentiation-19 (CD19) surface proteins and two of the therapies target B-cell maturation antigen (BCMA), both of which are found on the surface of B-cells [67,68]. CAR T-cell therapy has had remarkable efficacy in the management of hematological malignancies but little efficacy in the treatment of solid organ tumors. PRAME, as an intracellular antigen, raises concerns because traditional CAR T-cell therapies typically target surface proteins. Since PRAME is not expressed on the cell membrane but rather within the cell, targeting it directly through conventional CAR T-cell approaches is difficult. Despite this challenge, researchers are exploring novel strategies to overcome these obstacles. One such approach is the use of T-cell receptor (TCR)-engineered T-cells, which, unlike CAR T-cells, can recognize intracellular antigens presented on the cell surface by major histocompatibility complex (MHC) molecules [69,70]. This makes PRAME a promising target for TCR-based therapies rather than conventional CAR T-cells. TCR-engineered T-cells allows the immune system to target tumors more effectively, even in the context of solid tumors.

A clinical phase I/II trial is currently ongoing in mUM patients to assess the safety and activity of PRAME-TCR therapy (NCT02743611). Autologous T-cells (BPX-701) are modified to target PRAME on melanoma cells and include a biological safety switch that is controllable with rimiducid. Early reports of the project IMA203 (PRAME-TCR therapy) were presented at SITC 2021 [71]. Advancements in TCR-engineered therapies and modifications to CAR T-cell technology may provide a pathway to effectively target PRAME in solid tumors like melanoma. Continued research into optimizing these approaches is crucial to improve the feasibility of PRAME-targeted cellular therapies.

### 4.4. PRAME-CD3+ Bispecific Molecules (ImmTACs)

**Figure 3:**
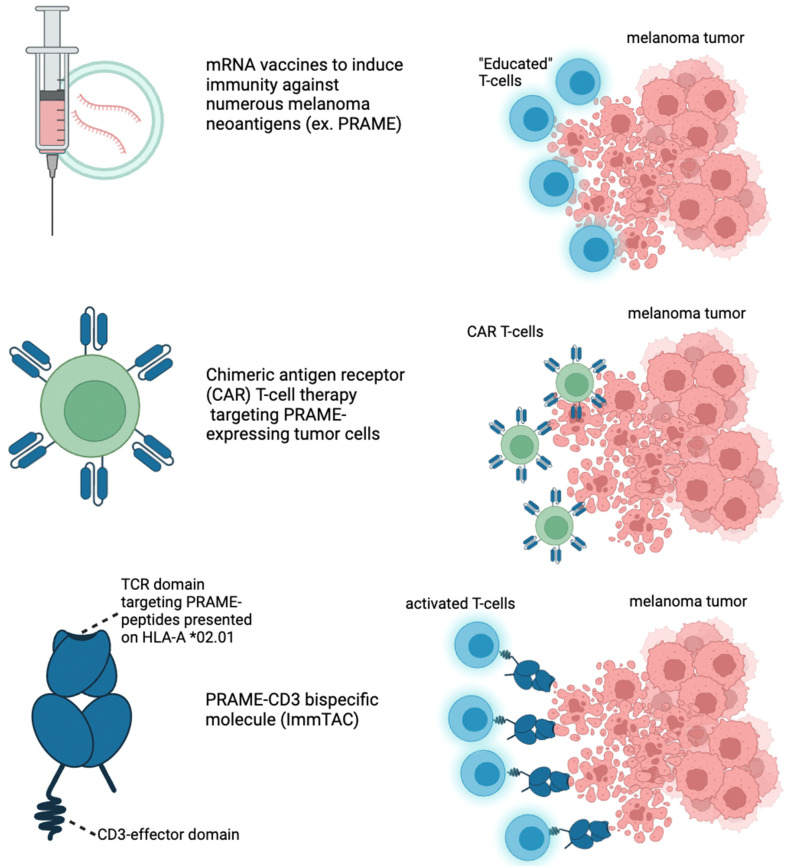
New immunotherapeutic strategies to target melanoma. mRNA vaccines can stimulate expression of neoantigens that are unique to patients’ tumor profiles. This stimulates “educated” T-cells to target and kill melanoma tumor cells. CAR T-cells are engineered to attack patients’ tumors based on engineered chimeric receptors; CAR-T therapy works by collecting a patient’s T-cells, genetically modifying them to express a chimeric antigen receptor (CAR) that can specifically recognize and bind to patients’ cancer cells, and then re-infusing these engineered T-cells back into the patient. Once inside the body, the modified T-cells can recognize and attack cancer cells that express the targeted antigen, leading to their destruction. This approach harnesses the immune system’s natural ability to fight cancer, with the CAR providing enhanced specificity and efficacy.

Brenetafusp (IMC-F106C) is an ImmTAC (Immune Mobilizing Monoclonal TCR Against Cancer) bispecific molecule developed by Immunocore (Figure 3, Table 3). It targets PRAME (Preferentially Expressed Antigen in Melanoma) while also pulling T-cells to the tumor directly. Brenetafusp is designed to redirect T-cells to recognize and kill PRAME-positive cancer cells. This molecule works by utilizing a soluble T-cell receptor (TCR) that binds to intracellular cancer antigens, like PRAME, with high specificity and affinity. It then recruits T-cells through an anti-CD3 effector function, activating the immune system to attack and destroy the cancerous cells. Brenetafusp has shown promising results in early clinical trials, both as a monotherapy and in combination with anti-PD1 therapies, for treating late-stage melanoma patients. In the phase 1 clinical trials of brenetafusp (IMC-F106C), an ImmTAC bispecific molecule targeting PRAME, for patients with late-line, immune checkpoint pre-treated cutaneous melanoma, the treatment demonstrated promising disease control. The results showed a 58% disease control rate and a median progression-free survival (PFS) of 4.2 months in PRAME-positive patients. The treatment was well tolerated, with manageable adverse events, primarily mild cytokine release syndrome (CRS) and rash. Additionally, 42% of ctDNA-evaluable, PRAME-positive patients had a molecular response, suggesting potential long-term benefits. Brenetafusp also showed better outcomes in earlier lines of therapy, and it was observed that T-cell fitness was associated with increased clinical activity. The combination of brenetafusp with anti-PD1 therapy, such as pembrolizumab, also demonstrated efficacy in patients with heavily pre-treated cutaneous melanoma. The ongoing phase 3 trial (PRISM-MEL-301) aims to further evaluate the efficacy of brenetafusp combined with nivolumab in first-line advanced melanoma patients.

### 4.5. MRNA Vaccines

PRAME is involved in immune evasion mechanisms, such as inhibiting retinoic acid signaling, which is essential for differentiation and apoptosis in malignant cells. Its role in immune modulation and melanoma progression makes it an ideal target for immunotherapies like mRNA vaccines [27,72,73]. Recent advancements in mRNA vaccine technology have made it possible to create personalized cancer vaccines that encode multiple tumor-specific antigens, including PRAME [74]. mRNA vaccines encoding PRAME antigens have been tested in preclinical or early-phase clinical trials for melanoma and other cancers [75,76]. Merck and Moderna’s mRNA-4157/V940 vaccine, developed in combination with pembrolizumab (Keytruda), is showing promising results as a new approach to melanoma treatment. This personalized mRNA-based cancer vaccine works by encoding up to 34 neoantigens, tailored to the unique mutational profile of a patient’s tumor (Figure 3, Table 3). The vaccine is designed to trigger an immune response specifically against these cancer-related mutations. Key recent developments include the results from the Phase 2b KEYNOTE-942 trial, which demonstrated that combining the mRNA-4157/V940 vaccine with pembrolizumab significantly improved recurrence-free survival (RFS) in patients with high-risk resected melanoma compared to pembrolizumab alone [77,78,79]. Importantly, this benefit was observed regardless of the tumor mutational burden, which suggests that this vaccine could be effective across different patient profiles. The safety profile of the combination therapy was consistent with known adverse effects, such as mild cytokine release syndrome and skin rash. Following these encouraging results, the vaccine has received FDA Breakthrough Therapy Designation, and a phase 3 trial has been initiated to further explore its efficacy in patients with melanoma. This combination therapy represents a potentially groundbreaking advance in melanoma treatment, offering a new personalized therapeutic option for patients at high risk of recurrence. As highly expressed as PRAME is within melanoma cells, it is likely that this approach will take advantage of PRAME along with other cancer-associated antigens.

### 4.6. Current Limitations in Immunotherapeutic Targeting of PRAME

The main limitations to the clinical use of PRAME-targeted vaccines and adoptive cell transfer are tumor heterogeneity, immune-suppressive tumor microenvironment (TME), negative thymic selection, and downregulation of HLA-1 molecules. In any one tumor site, there is often heterogeneity in the expression of cancer-specific antigens on HLA-1 molecules. In a melanoma tumor, for example, some cells may express PRAME on the surface while other cells do not. Several methods are being developed to sidestep this issue. As mentioned previously, PRAME expression is the result of hypomethylation of the promoter region for the PRAME gene in the nucleus of cells. By utilizing molecules that hypomethylate the PRAME gene promoter, PRAME expression in tumor cells can be artificially induced, thereby limiting the heterogeneity of antigen expression. Demethylating agents and histone deacetylase (HDAC) inhibitors like Decitabine, Sebularine, and DZNep could potentially be utilized as adjuvants for ACT and PRAME-targeted vaccines [5,8,10]. The second key limitation of PRAME-targeted immunotherapy is the immune-suppressive TME. Tumor cells are often recognized by CD8+ cells, but the cancer cells suppress the response through various mechanisms. Myeloid-derived suppressor cells (MDSCs), tumor-associated macrophages, and regulatory T-cells, recruited by tumor cells, secrete molecules promoting immunosuppression [66]. Another way tumor cells cultivate an immunosuppressive TME is through the production of checkpoint molecules. To prevent frequent autoimmune reactions, somatic cells produce cytotoxic T-lymphocyte-associated protein 4 (CTLA-4), programmed cell death protein 1 (PD-1), and programmed death ligand 1 (PD-L1), which signal to a T-cell to stop the release of cytotoxic enzymes. Tumor cells utilize these molecules to prevent their destruction by T-cells. To limit the immune suppression, combination therapies of cancer vaccines and checkpoint inhibitors like nivolumab and ipilimumab are being researched. Multiple preclinical and phase I trials have found increased efficacy compared to monotherapy. Several other trials exploring combination therapy are ongoing [56,80]. Another key limitation is the frequently observed lack of CD8+ proliferation secondary to negative thymic selection. Thymic selection of T-cells is a process where T-cells recognizing human antigens are destroyed to prevent autoimmunity. This allows T-cells recognizing foreign pathogens to proliferate and enter the circulation while T-cells recognizing human antigens are eliminated. Because members of the CTA family are expressed in normal germline tissues, it is likely that T-cells recognizing these endogenous antigens are eliminated to prevent autoimmunity [5]. The final limitation to discuss is the tumor cell’s ability to downregulate the expression of HLA-1 molecules, thereby preventing recognition by CD8+ cells. One possible solution is to induce the expression of HLA-1 molecules in tumor cells through the administration of MEK inhibitors, demethylating agents, and HDAC inhibitors [5,81]. Additional ways to circumvent tumor heterogeneity, immune-suppressive tumor microenvironment, negative thymic selection, and downregulation of HLA class 1 molecules should continue to be studied to improve the efficacy of immunotherapy in cancer patients.

## 5. Conclusions

PRAME is a remarkable protein with unique properties that aid in the diagnosis, prognosis, and treatment of melanoma. The selective presentation of PRAME on the HLA-1 molecules of tumor cells, not somatic cells, makes it a tumor-associated antigen of interest. Staining melanocytic lesions with PRAME IHC stains provides aid in accurate diagnoses with excellent sensitivity and specificity for melanoma versus benign melanocytes or dysplastic melanocytes. PRAME can also aid in margin assessment in melanoma excisions and in determining Breslow thickness for melanoma staging, particularly in superficial melanoma arising within a background of benign or dysplastic nevi. The utilization of PRAME as a marker for prognosis will likely become relevant as more biomarker trials are performed. Multiple studies demonstrated increased PRAME expression to be associated with Class II UM, the more aggressive subtype, and associated with stage III-IV cancers. Using PRAME staining in the cancer staging process could yield a more accurate prognosis, guiding clinicians to make better treatment decisions. Moreover, we highlighted the prospect of PRAME as a therapeutic target in immunotherapy. The literature for PRAME-targeted immunotherapy is not as developed as it is for other TAAs but is still promising. The limitations, including negative thymic selection, immune-suppressive TME, tumor heterogeneity, and downregulation of HLA-1 molecules, are significant barriers to progress in the field (Figure 3, Table 3). By improving our understanding of PRAME, we can better harness the power of immunotherapy and related modalities to prolong survival in patients with metastatic melanoma and, hopefully, detect melanoma earlier with strategies to prevent the development of metastases.

## Figures and Tables

**Table 1:** PRAME as a prognostic marker in melanoma and other cancers.

Cancer Type	Study	PRAME Expression Rate	Prognostic Outcome
Uveal Melanoma (Class 2)	Field et al., 2016 [40]	High in Class 2	Increased metastasis risk, worse prognosis
Mucosal Melanoma	Toyama et al., 2019 [41]	83.3%	Correlated with worse overall survival
Breast Cancer	Epping et al., 2008 [42]	N/A	Associated with decreased overall survival
Sarcomas	Iura et al., 2015 [43]	90% in myxoid liposarcoma	PRAME positivity associated with worse prognosis
Leukemia (ALL)	Zhang et al., 2017 [44]	N/A	Overexpression correlated with better outcomes in ALL
Hodgkin’s Lymphoma	Xu et al., 2020 [45]	N/A	PRAME overexpression associated with drug resistance

**Table 2:** PRAME in immunotherapy clinical trials.

Therapy Type	Phase	Target Cancer	Clinical Outcome
PRAME-targeted vaccine	I	Melanoma, NSCLC	CD4+ response but limited/no CD8+ response
PRAME-specific TILs	Preclinical	Melanoma, AML	36% (Melanoma) and 70% (AML) of patients showed PRAME-specific TILs
PRAME-CD3 bispecific molecules	III	Cutaneous Melanoma	58% disease control rate, promising PFS (4.2 months)
PRAME-CAR T-cells	Preclinical	Various cancers	Promising preclinical results but high risk of off-target toxicities

**Table 3:** Challenges in PRAME-targeted therapies.

Challenge	Description	Potential Solutions
Tumor Heterogeneity	Not all tumor cells express PRAME, leading to selective destruction of some but not all tumor cells.	Use of demethylating agents like Decitabine to induce PRAME expression across all tumor cells.
Immune-Suppressive Tumor Microenvironment (TME)	Tumor cells recruit immune-suppressive cells (e.g., T-regs, MDSCs) that block immune response.	Combination therapies with immune checkpoint inhibitors like anti-PD1 (nivolumab) and anti-CTLA-4 (ipilimumab).
Negative Thymic Selection	T-cells recognizing PRAME as “self” may be destroyed during thymic selection, reducing the immune response.	Further research into overcoming thymic selection with engineered T-cells or improved antigen presentation.
Downregulation of HLA-1 Molecules	Tumor cells reduce the expression of HLA-1 molecules, preventing recognition by CD8+ T-cells.	Use of MEK inhibitors or HDAC inhibitors to induce HLA-1 expression on tumor cells.
